# Influence of foot pain on frailty symptoms in an elderly population: a case-control study

**DOI:** 10.1590/1516-3180.2020.0492.R1.0802021

**Published:** 2021-05-21

**Authors:** Emmanuel Navarro-Flores, Ricardo Becerro-de-Bengoa-Vallejo, César Calvo-Lobo, Marta Elena Losa-Iglesias, Patricia Palomo-López, Victoria Mazoteras-Pardo, Carlos Romero-Morales, Daniel López-López

**Affiliations:** I MSc, PhD, DPM. Assistant Professor, Department of Nursing, Faculty of Nursing and Podiatry, University of Valencia; and Frailty Research Organized Group (FROG), University of Valencia, Valencia, Spain.; II RN, BSc, MLIS, DPM, DHL, PhD. Full Professor, Department of Physiotherapy and Podiatry, School of Nursing, Universidad Complutense de Madrid, Madrid, Spain.; III PT, MSc, PhD. Assistant Professor, Department of Physiotherapy and Podiatry, School of Nursing, Universidad Complutense de Madrid, Madrid, Spain.; IV RN, MSc, PhD, DPM. Full Professor, Department of Nursing and Stomatology, School of Health Sciences, Universidad Rey Juan Carlos, Madrid, Spain; V MSc, PhD, DPM. Senior Lecturer, Department of Nursing, University Center of Plasencia, Universidad de Extremadura, Spain; VI RN, MSc, PhD. Assistant Professor, Department of Nursing, Physiotherapy and Occupational Therapy, School of Physiotherapy and Nursing, University of Castilla-La Mancha, Toledo, Spain.; VII PT, MSc, PhD. Senior Lecturer, Department of Sport Sciences, School of Sport Sciences, Universidad Europea de Madrid, Villaviciosa de Odón, Madrid, Spain.; VIII MSc, PhD, DPM. Senior Lecturer and Researcher, Health and Podiatry Group, Department of Health Sciences, School of Nursing and Podiatry, Universidade da Coruña, Ferrol, Spain.

**Keywords:** Frail elderly, Aged, Surveys and questionnaire, Foot deformities, Foot diseases, Frailty, Elderly, Foot disorders, Questionnaire

## Abstract

**BACKGROUND:**

Frailty is a condition that can increase the risk of falls. In addition, foot disorders can negatively influence elderly people, thus affecting their condition of frailty.

**OBJECTIVE:**

To determine whether foot pain can influence a greater degree of frailty.

**DESIGN AND SETTING:**

Cross-sectional descriptive study conducted at the University of Valencia, Valencia, Spain.

**METHODS:**

A sample older than 60 years (n = 52), including 26 healthy subjects and 26 foot pain patients, was recruited. Frailty disability was measured using the 5-Frailty scale and the Edmonton Frailty scale (EFS).

**RESULTS:**

There were statistically significant differences in the total EFS score and in most of its subscales, according to the Mann-Whitney U test (P < 0.05). In addition, foot pain patients presented worse scores (higher 5-Frailty scores) than did healthy patients, regarding matched-paired subjects (lower EFS scores). With regard to the rest of the measurements, there were no statistically significant differences (P > 0.05). The highest scores (P < 0.001) were for fatigue on the 5-Frailty scale and the EFS, and for the subscale of independence function in EFS.

**CONCLUSIONS:**

These elderly patients presented impairment relating to ambulation and total 5-Frailty score, which seemed to be linked to the presence of frailty syndrome and foot disorders.

## INTRODUCTION

Aging and chronic illness processes like hyperglycemic disease, musculoskeletal disorders and heart processes can produce frailty syndrome. Consequently, these degenerative processes produce alterations that can affect mental and general health.^1^ For example, aging and frailty can affect gait speed and increase the risk of falling due to balance alterations.^2^^,^^3^^,^^4^ Furthermore, the presence of frailty symptoms affects health-related quality of life (HQoL)^5^ in this population group.

Frailty syndrome can be defined as a group of health alterations that can affect several aspects of the aging process. These alterations are a consequence of a dynamic process that has psychological, biological and social characteristics and which reduces health status.^6^ The frequency of frail states among people older than 65 years has been estimated to be between 4% and 59.1%.^7^

Among foot conditions in the elderly population, foot disorders and diseases are present most frequently in the frail population group, comprising approximately 25% of foot disorders.^8^^,^^9^

Consultations with general practitioners relating to ankle and foot conditions involving osteoarticular pain account for 8% of all consultations.^10^ Accordingly, distress caused by pain may raise this percentage. Elderly people have characteristic foot complaints that can be likened to bigger disorders.^11^ Foot health forms part of health-related quality of life (HQoL)^12^ and poor foot health gives rise to a risk of falls.^13^^,^^14^

Clinimetric health questionnaires for measuring the degree of frailty degree are necessary in order to correlate foot disabilities and the level of frailty.

The 5-Frailty scale is a questionnaire of five items that was set up to be self-administered.^6^ Respondents can provide affirmative or negative answers, and one point is given for a positive response. Thus, the total score can range from zero to five points, and subjects are classified as robust if the score is zero points, pre-frail with one to two points, or frail with three or more points. These classifications represent the subjects’ respective tiredness, resistance, ambulation, disease and weight loss.

Tiredness is evaluated by asking subjects if they feel tired; resistance is determined from their self-reported capacity to climb stairs; ambulation consists of their self-reported ability to move around; illness is determined as the presence of more than five out of a total of eleven pathological conditions, including cardiovascular diseases and diabetes; and loss of weight as a reduction of 5% during the last year.^15^

The Edmonton Frailty Scale (EFS) assesses nine subscales: 1) cognitive, 2) general health status, 3) independence, 4) social support, 5) pharmacological treatment, 6) feeding, 7) mood, 8) continence and 9) functional performance, using eleven questions. The maximum score is 17 and represents the highest degree of frailty.^16^ A score of between zero and four does not represent frailty; scores of five to six represent apparently vulnerability, scores of seven to eight represent mild frailty, scores of nine to ten represent moderate frailty and scores of eleven or more represent severe frailty.^17^

No study has yet correlated the scores of the EFS and the 5-Frailty scale. Therefore, the goal of the present study was to correlate the subscales of the EFS and 5 Frailty Scale among elderly people with and without foot disorders.

We were unable to find any references in the literature to the frailty status of elderly people with foot pain. Therefore, our hypothesis was that differences in levels of frailty exist among elderly people with foot pain.

## OBJECTIVE

The objective of this study was to determine whether foot pain can influence a greater degree of frailty.

## METHODS

This study was developed in Spain. We recruited elderly patients at a medical center, a rehabilitation service and a podiatry clinic, and all survey data were collected between October 2019 and January 2020. We obtained signed informed consent statements from all subjects. The observations for this study were made in accordance with the Strengthening the Reporting of Observational Studies in Epidemiology (STROBE) statement.^18^

### Sample size calculation

To calculate the sample size, the G*Power 3.1.9.2 software (Heinrich-Heine-Universität Düsseldorf; Düsseldorf, Germany) was used. The following assumptions were made: differences between two independent means would be tested; the hypothesis was two-tailed; a large effect size of 0.8 was used; the α error was taken to be 0.05, with a 95% confidence interval; the β error was taken to be 20%; and the 1-β power analysis was taken to be 0.80. From this, a total sample size of 52 subjects was determined, with 26 in each group.

### Participants

Before beginning the study, approval for conducting this study was obtained from the Ethics Committee of the University of Extremadura, Badajoz, Spain, under the registration number 1/2020, with the approval date March 16, 2020.

Informed consent was obtained from each participant after the purpose and process of the study had been explained to them. The participants were given an assurance that their information would remain confidential. The fact that their participation was entirely voluntary was also highlighted.

The criteria for including patients were that they needed to be elderly people (60 years of age or over) who presented foot pain during the last six months due to toe or foot deformities (but without wounds), regardless of their origin or cause, with a score of more than five points on a visual analogue scale (VAS); and were able to communicate orally and provide written informed consent. VAS scores above five points, i.e. from moderate to severe, showed intraclass correlation coefficient reliability of 0.870.^19^

The exclusion criteria were presence of major neurocognitive disorder, failure to answer the initial identification questions, inability to understand the rules of participation and refusal to participate in the study (through not signing the consent statement).

To recruit volunteer participants, we posted recruitment flyers in places within an elderly people’s center where people would gather together. We also addressed groups of elderly people at the center to invite them to contact us if they were willing to participate in the study. Once a potential participant expressed interested, a cognitive function evaluation was performed by a gerontological nurse practitioner (GNP), to establish the cognitive eligibility of the participant. Following the evaluation by the GNP, the investigators explained the study procedures in detail to the participant.

The interviews comprised questions on general health status, sociodemographic characteristics (sex, age, body mass index, height and weight) and comorbidities (e.g. anxiety, depression, diabetes, obesity, osteoarticular diseases, vascular disorders or kidney illness). Data on comorbidities were collected from the patients’ medical records. Furthermore, specific items relating to foot pain, such as current treatment or presence of foot deformities, were assessed by a senior podiatry physician (ENF)

In this study, a total of 65 elderly people expressed interest in participating in the study, and all of them met the cognitive requirements. The participants all attempted to complete the survey questionnaires. Subsequently, all the survey questionnaires were analyzed for this study. However, 14 of them were excluded due to incomplete answers. For participants who were not able to read the questionnaires due to vision problems, the investigators read the questions aloud and marked the participants’ answers on the questionnaires. The participants took about 15 minutes to complete the questionnaires. They did not receive any compensation for their participation in the study.

### Evaluation of frailty

The EFS was designed to measure frailty on nine subscales: cognitive, general health status, independence, social support, pharmacological treatment, feeding, mood, continence and functional performance.^16^^,^^21^ Its total scores range from 0 to 17, and higher scores indicate more frailty. The scores were classified into three degrees of frailty.^21^ Subjects who scored 0-5 points were designated as non-frail. Those who obtained 6-11 points were designated as ostensibly susceptible to frailty. Those who scored 12-17 points were designated as frail. The questionnaire only took 15 minutes to complete.

The participants also completed the 5-item Frailty scale.^22^ This scale measures five subscales: tiredness, resistance, ambulation, disease and weight loss. The frailty subscale scores each range from 0 to 5, and higher scores indicate more frailty. Participants who scored between three and five were considered to be frail; those who scored one or two were considered to be pre-frail, and those who scored zero points were considered to be non-frail.^17^

### Statistical analysis

All variables were normally distributed, as determined by the Kolmogorov-Smirnov test (P > 0.05).

Among the quantitative variables, nonparametric data were described in terms of their median, interquartile range (IR) and 95% confidence interval (CI). Parametric data were described using their mean, standard deviation (SD) and minimum and maximum (range) values.

A comparison of the quantitative data between men and women for the different questionnaire subscales of the EFS and the 5-Frailty scale was conducted, and significant differences were checked using an independent Student t test. Non-normal data were analyzed using the Mann-Whitney U test.

All analyses were considered statistically significant when the P-value was < 0.05 with a 95% CI. Statistical analyses were developed using the SPSS software, version 26.0 (SPSS, Chicago, IL, United States).

## RESULTS

### Descriptive data and sociodemographic data

Age, height, weight and body mass index were shown to have normal distribution (P > 0.05). On the other hand, none of the items from the 5-Frailty test or EFS showed normal distribution (P < 0.05).

The sample included 52 subjects whose mean age was 77.47 ± 10.69 years. The study subjects included 26 with foot pain (50.00%) and 26 healthy subjects (50.00%). Table 1 shows the sociodemographic characteristics. There were no statistically significant differences (P > 0.05) between the foot pain patients and the healthy individuals regarding the sociodemographic characteristics of age or body mass index.

**Table 1.** Descriptive and sociodemographic data of the sample

Demographic and descriptive data	All participants n = 52 Mean ± SD (95% CI)	Foot pain group n = 26 Mean ± SD (95% CI)	Healthy group n = 26 Mean ± SD (95% CI)	P-value^*^
Age (years)	76.80 ± 9.99 (74.34-79.26)	72.50 ± 7.83 (67.52-77.47	77.75 ± 10.23 (74.96-80.55	0.088
Weight (kg)	62.27 ± 11.60 (59.42-65.12)	67.41 ± 15.29 (57.69-77.13)	61.12 ± 10.45 (58.27-53.98	0.170
Height (m)	1.60 ± 0.08 (1.58-1.62)	1.64 ± 0.09 (1.58-1.70)	1.59 ± 0.07 (1.57-1.62)	0.083
Body mass index (kg/m^2^)	24.02 ± 3.75 (23.10-24.95)	24.75 ± 4.45 (21.92-27.59)	23.86 ± 3.60 (22.88-24.85)	0.612

Comparison of demographic characteristics of the total sample (all participants), subjects with foot pain and healthy subjects matched with normalized reference values.

^*^Mean ± standard deviation (SD), range (minimum-maximum) and Student’s t test for independent samples were applied; In all the analyses, P < 0.05 (with a 95% confidence interval, CI) was considered statistically significant.

### Edmonton Frail Scale and 5-Frailty scale distribution

As shown in Table 2, the 5-Frailty scale scores did not manifest any statistically significant difference (P > 0.05) for subscales or total scores between the foot pain and healthy groups. Furthermore, the distribution of EFS scores is shown in Table 3. The EFS subscales did not show any statistically significant differences (P > 0.05).

**Table 2.** Comparisons of 5-Frailty scale scores between foot pain and healthy groups

Frailty Scale Domains	Foot pain group n = 26 Mean ± SD (95% CI) Median (IR)	Healthy group n = 26 Mean ± SD (95% CI) Median (IR)	P-value
Fatigue	0.91 ± 0.30 (0.71-1.11) 1.00 (0.00)	0.45 ± 0.50 (0.29-0.61) 0.00 (1.00)	0.007
Resistance	0.36 ± 0.50 (0.02-0.70) 0.00 (1.00)	0.50 ± 0.50 (0.34-0.66) 0.50 (1.00)	0.427
Ambulation	0.55 ± 0.52 (0.19-0.90) 1.00 (1.00)	0.43 ± 0.50 (0.26-0.59) 0.00 (1.00)	0.481
Illness	0.64 ± 0.50 (0.30-0.98) 1.00 (1.00)	0.38 ± 0.49 (0.22-0.53) 0.00 (1.00)	0.125
Weight loss	0.64 ± 0.50 (0.30-0.98) 1.00 (1.00)	0.53 ± 0.50 (0.36-0.69) 1.00 (1.00)	0.515
Total Frailty Scale	3.27 ± 1.34 (2.37-4.18) 4.00 (2.00)	2.28 ± 1.48 (1.80-2.75) 2.00 (2.00)	0.059

CI = confidence interval; IR = interquartile range. Mann-Whitney U tests were used. In all the analyses, P < 0.05 (with a 95% confidence interval) was considered statistically significant.

**Table 3.** Comparisons of Edmonton Frail Scale scores between foot pain and healthy groups

Edmonton Frail Scale Domains	Foot pain group n = 26 Mean ± SD (95% CI) Median (IR)	Healthy group n = 26 Mean ± SD (95% CI) Median (IR)	P-value
Cognition	0.91 ± 0.70 (0.44-1.38) 1.00 (1.00)	0.75 ± 0.63 (0.55-0.95) 1.00 (1.00)	0.488
General health status 2A	0.73 ± 0.64 (0.29-1.16) 1.00 (1.00)	0.65 ± 0.62 (0.45-0.85) 1.00 (1.00)	0.718
General health status 2B	1.09 ± 1.04 (0.39-1.79) 1.00 (2.00)	0.75 ± 0.84 (0.48-1.02) 1.00 (1.00)	0.302
Functional independence	1.18 ± 0.98 (0.52-1.84) 1.00 (2.00)	0.45 ± 0.74 (0.21-0.69) 0.00 (1.00)	0.011
Social support	0.64 ± 0.50 (0.30-0.98) 1.00 (1.00)	0.45 ± 0.59 (0.28-0.64) 0.00 (1.00)	0.238
Medication use 5A	0.73 ± 0.46 (0.41-1.04) 1.00 (1.00)	0.58 ± 0.50 (0.41-0.74) 1.00 (1.00)	0.364
Medication use 5B	0.55 ± 0.52 (0.19-0.90) 1.00 (1.00)	0.55 ± 0.50 (0.39-0.71) 1.00 (1.00)	0.979
Nutrition	0.82 ± 0.40 (0.55-1.09) 1.00 (1.00)	0.63 ± 0.49 (0.47-0.78) 1.00 (1.00)	0.233
Mood	0.55 ± 0.52 (0.19-0.90) 1.00 (1.00)	0.55 ± 0.50 (0.39-0.71) 1.00 (1.00)	0.979
Continence	0.27 ± 0.46 (0.04-0.59) 0.00 (1.00)	0.43 ± 0.50 (0.26-0.59) 0.00 (1.00)	0.364
Functional performance	1.00 ± 0.63 (0.58-1.42) 1.00 (0.00)	1.08 ± 0.65 (0.87-1.28) 1.00 (1.00)	0.728
Total Edmonton Frail Scale	8.09 ± 5.43 (4.44-11.74) 9.00 (9.00)	6.69 ± 4.33 (5.26-8.04) 6.00 (7.00)	0.477

CI = confidence interval; IR = interquartile range. Mann-Whitney U tests were used. In all the analyses, P < 0.05 (with a 95% CI) was considered statistically significant.

## DISCUSSION

The two scales could be correlated, which confers concurrent validity on each subscale, as used in recent studies, and sustains application of the 5-Frailty score as an acceptable measurement relating to aspects of frailty such as ambulation, illness or weight loss. This can be considered to be an advantage in relation to other frailty scales that have been adapted for use in Spanish to evaluate specific aspects of frailty, like the Frailty Trait Scale (FTS).^23^

The frequency of occurrence of frailty factors, especially among elderly people, requires adequate measurement of frailty scores. Our research has shown that frailty relating to biomechanical parameters like gait speed presents lower scores. It has also been shown that women have higher degrees of frailty than do men, when both have foot pain.^24^^,^^25^ Our present results are along the same lines as in previous studies relating to frailty and foot disorders, which showed similar results relating to frailty scores and foot disorders.^26^^,^^27^

Moreover, balance disorders have been shown to increase frailty scores, and the our results coincide with those from previous studies.^28^^,^^29^ Thus, altered walking ability and balance are characteristics of frailty. Specifically, women with foot disorders exhibited higher frailty scores than men, with the exception of the EFS mood subscale, which seems be related to the existence of foot disorders and the aging process. Our results were similar to those of other authors.^5^^,^^8^

Future studies should incorporate all other foot risk factors related to frailty syndrome. Although a frailty score is determined through the EFS,^25^^,^^30^ the Geriatrician’s Clinical Impression of Frailty (GCIF) has also been used in a cohort of older acute patients.^31^

Several limitations of this study need to be taken into account. A population from different areas might be useful to improve the strength of this study.

In the present study, it was only determined whether foot pain could influence a greater degree of frailty. We found that foot pain does not affect frailty.

Although gait disorders, balance alterations and the risk of falling are very common among frail people,^2^^,^^4^ studies like the present one should also be developed for other population groups, in order to determine their degree of frailty. For example, widows usually have higher frailty scores due to psychosocial factors.^30^^,^^32^^,^^33^

Furthermore, selective sampling can cause bias. For this reason, use of randomized sampling should be considered in future studies.

Lastly, the correlation between different foot disorders, including several genetic and acquired or traumatic alterations and chronic illnesses, was not studied here because our population was not suitable for developing these comparisons. We therefore suggest that future research should be conducted on different pathological conditions of the feet.

## CONCLUSIONS

Foot pain greater than five points on the 5-Frailty score scale seemed to be linked to the presence of frailty syndrome and foot disorders, especially the score relating to ambulation.
